# Evaluation the Impact of Hormonal Fluctuations During the Menstrual Cycle on the Performance of Female Athletes—Systematic Review

**DOI:** 10.3390/muscles4020015

**Published:** 2025-05-19

**Authors:** Ainize Elorduy-Terrado, Gema Torres-Luque, Krizia Radesca, Guillermo Muñoz-Andradas, Marisa Saenz-Bravo, Diego Domínguez-Balmaseda

**Affiliations:** 1 Department of Real Madrid Graduate School, Universidad Europea de Madrid, 28670 Madrid, Spain; ainize16@gmail.com (A.E.-T.); krizia.radesca@universidadeuropea.es (K.R.); guillermo.munoz@universidadeuropea.es (G.M.-A.); marisa.saenz@universidadeuropea.es (M.S.-B.); 2 Faculty of Humanities and Science Education, University of Jaén, 23071 Jaén, Spain; gtluque@ujaen.es

**Keywords:** menstrual cycle, estrogen, progesterone, female athletes, eumenorrheic, performance

## Abstract

This systematic review aims to evaluate the impact of hormonal fluctuations during the menstrual cycle on the performance of female athletes. Methods: Following PRISMA guidelines, a comprehensive search was conducted in Scopus, Web of Science, and PubMed databases using the keywords: (“Menstrual cycle”) AND (“performance” OR “female athlete” OR “sport” AND NOT “male”); AND NOT (“contraceptive”). Inclusion criteria focused on original studies published between 2013 and 2023, in English or Spanish, involving eumenorrheic female athletes without menstrual disorders or oral contraceptive use. The studies were critically assessed using the McMaster scientific review method. Results: Thirteen eligible articles were reviewed, comprising a total sample of 152 athletes. Significant findings include increased flexibility during the ovulatory phase and enhanced aerobic and anaerobic capacities in the luteal phase. Additionally, the menstrual and premenstrual phases notably influenced aerobic and anaerobic capacities, performance perception, symptomatology, and exercise-induced muscle damage. Conclusion: Hormonal fluctuations can impact female athletes’ performance. However, further research is warranted due to inconsistent results stemming from variations in cycle phases studied, lack of standardized methodologies, small sample sizes, and short observation periods.

## 1. Introduction

Sports represent a global social phenomenon that transcends cultural boundaries, creating shared experiences and fostering enthusiasm worldwide [1]. However, societal challenges, such as gender inequality, have historically been mirrored in sports, with women being excluded from participation for many years.

In recent times, the growth and professionalization of women’s sports have led to an increase in research focusing on female physiology, particularly the effects of sex hormones on athletic performance [2]. Although findings vary, there is broad agreement that biological differences between men and women—particularly the influence of steroid hormones such as progesterone (P4) and estradiol (E2)—significantly impact female athletes’ physiological responses to exercise [3].

The menstrual cycle, which typically lasts 28 days in eumenorrheic women, is regulated by interactions among hypothalamic, pituitary, and ovarian hormones [4]. The secretion of P4, follicle-stimulating hormone (FSH), luteinizing hormone (LH), and E2 occurs in distinct phases, resulting in hormonal fluctuations that influence physical performance and metabolic requirements [5]. These fluctuations can affect performance metrics and mood throughout the cycle, with E2 and P4 playing pivotal roles in these variations [6].

For instance, some studies [7] indicate that premenstrual symptoms during the late luteal phase—such as abdominal discomfort and mood changes—negatively impact performance perception. Additionally, Solli et al., (2020) [8] found that 20% of athletes modify their training routines or use analgesics during the early follicular phase to manage symptoms.

Performance parameters such as strength fluctuate, with increases observed during the luteal and ovulatory phases, while declines in strength and heightened fatigue are noted during the late luteal and early follicular phases [9,10,11]. Research on endurance has produced inconsistent results, with minor reductions in anaerobic capacity during the late follicular phase and lower aerobic capacity during the late luteal phase [12].

Furthermore, athletes with menstrual dysfunctions or delayed menstruation face an elevated risk of injury. In sports such as soccer, 88% of muscular and tendinous injuries occur during ovulation, likely due to elevated levels of estrogen and relaxin, which increase the risk of anterior cruciate ligament (ACL) injuries [13,14].

While there is evidence suggesting that hormonal fluctuations influence performance, the results remain inconsistent, underscoring the necessity of further research to achieve a more precise understanding [15]. Consequently, this study seeks to analyze the existing literature on the impact of hormonal fluctuations during the menstrual cycle on female athletes’ physical performance parameters over the past decade (2013–2023).

## 2. Methods

The present systematic review follows the guidelines established by PRISMA (Preferred Reporting Items for Systematic Reviews and Meta-Analyses), recommended for conducting systematic reviews and meta-analyses [16], and is registered on the platform https://www.crd.york.ac.uk/PROSPERO/ (accessed on 22 May 2024).

**Figure 1:**
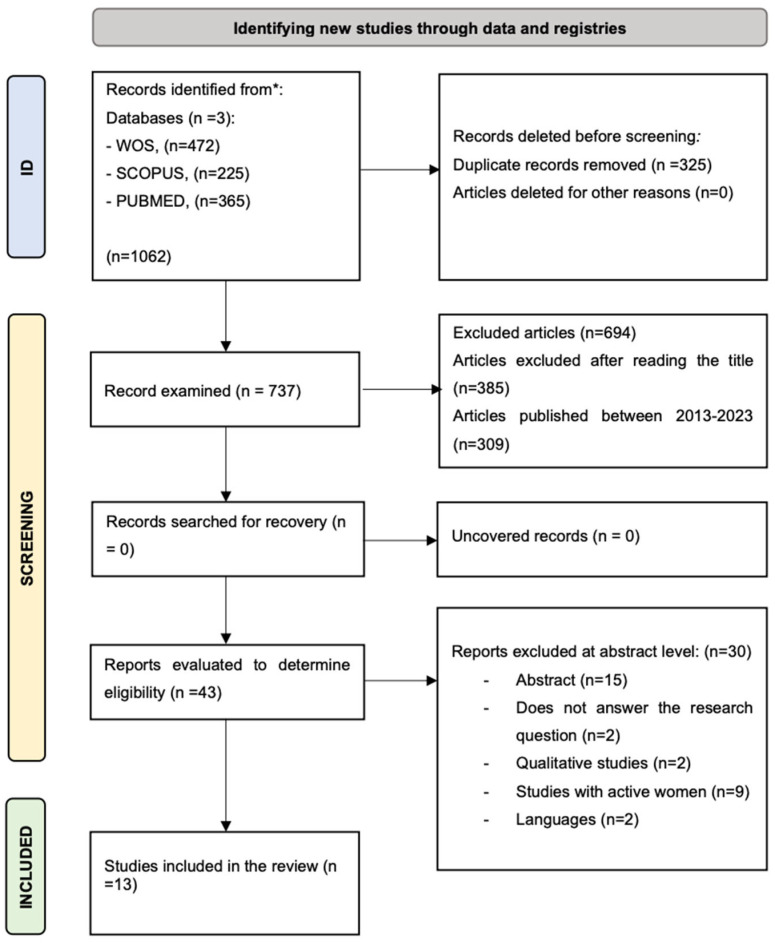
PRISMA 2020 Flow Diagram for New Systematic Reviews.

The systematic review follows a strategy for searching original articles. To identify eligible articles for the study, a search was conducted in the main databases: Scopus, Web of Science, and PubMed, using a search strategy that includes Boolean operators with the established search terms: (“menstrual cycle”) AND (“performance” OR “female athlete” OR “sport” AND NOT “male”); AND NOT (“contraceptive”) (Figure 1).

No restrictions were applied in the advanced search, and all studies published in the main database categories were included, incorporating all those indexed in the Core Collection of Web of Science.

### 2.1. Study Selection

The selection of studies for this systematic review follows the PICO criteria. Studies involving eumenorrheic female athletes participating in any sport [P] that assessed physiological and performance variables [I] in relation to the different phases of the menstrual cycle [C] were included to determine the significant effects of the menstrual cycle phases on athletic performance [O]. All articles that met the inclusion and exclusion criteria outlined in Table 1 were individually assessed using the McMaster form.

### 2.2. Inclusion and Exclusion Criteria

Data extraction from eligible articles is classified in a table that includes the following: (1) authors and year; (2) study design; (3) observation period; (4) sport; (5) category; (6) sample size; (7) mean age; (8) menstrual cycle measurement; (9) phases analyzed; (10) performance parameters and tests; (11) subjective variables; (12) main conclusions. Data from the included studies were recorded in a spreadsheet designed for this review.

The studies included in this systematic review were assessed using the McMaster form, which aims to evaluate the methodological quality and risk of bias of the research [17]. Sixteen items were evaluated to determine whether the studies met specific criteria, with scoring conducted using two indicators: “YES = 1 point” or “NO = 0 points”, except for items 6 and 13, for which the response “Not applicable” could also be selected.

Finally, the total score of the studies classifies the articles into five categories: poor methodological quality (P with ≤ 8 points); acceptable methodological quality (A with 10-11 points); good methodological quality (G with 12–13 points); very good methodological quality (VG with 14-15 points); and excellent methodological quality (E with ≥ 16 points).

## 3. Results

The systematic review includes a total of 13 scientific articles (Table 2), with significant heterogeneity in both the characteristics of the athletes and the performance tests conducted, as multiple sports disciplines were included.

The table summarizes studies on how the menstrual cycle affects female athletic performance. Different cycle phases were analyzed in sports like soccer, basketball, and athletics using physical tests and biological analyses. Some studies found reduced performance or well-being in certain phases, while others found no significant differences.

The sample of the included studies comprises a total of 152 athletes, all of whom have eumenorrheic menstrual cycles, without the need for contraceptive use of any kind among the total participants. Ten articles focus on team sports, involving 118 athletes, including 75 soccer players [20,21,24,25,27,30], 18 handball players [26,29], 14 futsal players [23], and 11 basketball players [28]. Three articles focus on individual sports, with a total of 34 women: 13 track and field athletes specializing in 800 m and 1500 m distances [31], 13 triathletes [22], and 8 judokas [32].

The duration of the studies ranges from the analysis of an entire menstrual cycle, with one month of research [21,22,26], to durations of two months [31], three months [20,27,32], and even three years [30]. The main phases analyzed focus on the follicular and luteal phases, determined using tools such as blood tests, urine tests, and records in apps or calendars.

The main variables examined include the phases of the menstrual cycle and sport-specific performance parameters, with performance tests specific to each sport, competition measurements, and even subjective variables, as detailed in Table 3 and Table 4.

All studies included in the systematic review investigate the follicular phase of the menstrual cycle, and most of them distinguish between the early follicular phase, including menstruation, and the late follicular phase [20,21,25,27,29,30,31], while five studies analyze it as a whole [23,24,26,31,32]. The luteal phase is also analyzed by the authors; five of them [20,22,29,30,32] focus on the mid-luteal phase, whereas one of them studies the premenstrual phase [26]. In addition, two studies [25,31] also incorporate the ovulatory phase in their research, with Shakhlina and colleagues (2016) [31] investigating all stages of the menstrual cycle.

### 3.1. Influence of the Menstrual Cycle

Most studies included in this systematic review [20,21,22,23,25,26,27,29,31,32] assess athletes’ physical performance by analyzing key attributes such as lower limb strength, endurance, speed, flexibility, and agility. However, three studies [24,28,30] take a different approach, focusing on match-related statistics, distance covered, and game intensity, while also considering contextual factors that provide a more comprehensive understanding of the results. According to the authors, factors such as match outcome and opponent strength may influence performance metrics, potentially affecting the final conclusions of these investigations.

### 3.2. Impact of Menstrual Cycle on Game-Related Variables

Three studies [24,28,30] examined the impact of menstrual cycle phases on various performance parameters during matches. Two studies [24,30] focused on soccer players, analyzing total distance covered and match intensity, while the third study [28], conducted on basketball players, assessed performance index rating, rebounds, shooting accuracy, turnovers, goals per minute (measured through PlayerLoad), and subjective exertion variables.

For match intensity analysis, one study [24] classified intensity into four zones (low, high, very high, and sprint), distinguishing between high-intensity bouts and sprints. A statistically significant increase (*p* < 0.05) in distance covered in zone 3 (high-intensity) was observed during the luteal phase compared to the follicular phase, while no significant differences were found in other variables. Similarly, Igoinin’s study [30] reported a statistically significant increase (*p* < 0.001) in the number of sprints during the luteal phase, alongside a significant decrease in total distance covered at moderate and high speed during the follicular phase (*p* < 0.05). In contrast, Gasperi’s study [28] found that shooting accuracy and rebounds were significantly higher (*p* < 0.05) during the follicular phase compared to the luteal phase.

### 3.3. Impact of the Menstrual Cycle on Muscle Strength

Seven studies [20,21,22,25,26,27,29] evaluated lower limb muscle strength across different menstrual cycle phases.

Various tests were used to assess lower limb strength, with the most common being the countermovement jump (CMJ) [20,25,29], which measures power output. Other jump-based tests included the five-jump test (5JT) [21], the hop test, and the squat jump [27]. Additionally, the half-squat test [22] was used, though no significant differences were observed between phases.

Two studies assessed explosive strength through short-duration, high-intensity runs: Tounsi [21] used the repeated-sprint shuttle ability (RSSA) test, while Graja [26] applied the repeated sprint effort (RSE) test. Graja’s study also included a maximal voluntary contraction (MVC) test for the vastus medialis, vastus lateralis, and rectus femoris muscles post-RSA, evaluating neuromuscular efficiency and muscle damage across menstrual phases.

Graja [26] found significant differences in the premenstrual phase compared to the follicular and luteal phases, reporting lower peak power in RSE, reduced neuromuscular efficiency in MVC, and increased muscle damage (*p* < 0.05). Higher MVC was observed in the follicular phase compared to the luteal and premenstrual phases. Additionally, peak power significantly decreased in the last two sprints of the luteal phase versus the follicular phase (*p* < 0.05), though RSA deceleration remained similar across phases, except for lower RSA deceleration in the follicular phase compared to the premenstrual phase (*p* < 0.05).

No studies on power tests showed statistically significant differences. However, one study [21] found a significant increase (*p* < 0.001) in 5JT performance during afternoon sessions compared to morning sessions. Similarly, no significant differences were observed in the 20-m [25] or 40-m [27] sprints used to assess explosive strength in short-duration runs.

### 3.4. Impact of Menstrual Cycle on Aerobic and Anaerobic Capacity

Six of the studies examine the aerobic and anaerobic capacity of the athletes, applying tests such as the Yo-Yo test [20,21], Balke maximal test [23], 4 · 400 m test and PWC170 [31], and finally, Wingate and SJFT [32], finding statistically significant differences in several studies.

On the one hand, Ross and colleagues [20] evaluated maximal aerobic capacity using the Yo-Yo IET test, in which they observed a significant increase (*p* < 0.05) in pre-exercise heart rate and a lower total distance covered, with results approaching significance (*p* = 0.07), during the mid-luteal phase compared to the early follicular phase. Additionally, three and five minutes after the test, they demonstrated higher lactate concentrations during the follicular phase compared to the luteal phase, with significant results between phases (*p* < 0.05). Conversely, the study conducted by Shakalina and colleagues [31] reported a greater physical working capacity in the PWC170 test during the luteal phase compared to the follicular and ovulatory phases (*p* < 0.05), which aligns with another study [33], where better results were obtained for maximal aerobic capacity, with a statistically significant increase in VO2Max and test duration (*p* < 0.05) during the luteal phase.

On the other hand, the same study [31] found differences in the results of anaerobic capacity, evaluated by a series of 4x400m runs, during the menstrual, premenstrual (early follicular), and ovulatory phases, compared to the late follicular and mid-luteal phases. Specifically, they reported the following: firstly, a statistically significant increase in time during the menstrual and premenstrual phases, indicating lower performance in the series conducted during these phases; secondly, an increase in blood lactate during the menstrual and ovulatory phases; and lastly, an increase in average heart rate after the test during the menstrual, premenstrual, and ovulatory phases.

However, no significant differences were found in the tests conducted by Tounsi and colleagues [21] for the evaluation of VO2Max using the YYIRT1 test, as well as in the Wingate and SJFT tests in Stefanosky’s study [32].

### 3.5. Impact of the Menstrual Cycle on Agility and Flexibility

Other authors decided to include different tests in their study, beyond the analysis of conditional physical capacities, covering the adaptation of other abilities during the phases of the menstrual cycle. On the one hand, Sanchez and colleagues [27] implemented the V-cut test to evaluate the change in direction speed in soccer players, showing a 2.7% improvement in agility during the follicular phase, although without significant differences in the results. On the other hand, Campa and colleagues [25] included the sit and reach test in their analysis, evaluating the flexibility of soccer players during the early follicular and ovulatory phases, showing a decrease in flexibility during the early follicular phase (*p* < 0.05).

### 3.6. Impact of Menstrual Cycle on Physiological Variables

The five studies that include an analysis of subjective variables in their research involve athletes from team sports, using different rating techniques. Two of them [25,26] conducted this analysis using the Hooper Index; others [20,28], meanwhile, used the RPE; and lastly, one of them [29] employed the SAM and VAS scales.

Only one of the studies [27] demonstrates a relationship between perceived well-being in female athletes and the menstrual cycle, showing a decrease during the early follicular phase (specifically during menstruation) and in the luteal phase compared to the late follicular phase, with statistically significant differences observed between phases (*p* < 0.05). In the remaining four studies, no significant differences were found in subjective variables across phases. However, in one of the studies [28], a better shooting efficiency was associated with better results in RPE and TQRpre during the follicular and luteal phases, indicating that better perceived well-being in athletes is related to shooting efficiency.

### 3.7. Methodological Quality of the Studies

The methodological quality of the thirteen studies evaluated using the McMaster Critical Review Form ranged from a minimum quality of 68.75% to a maximum of 100.00%, with total scores ranging from 11 to 16 points. Some studies were classified as “excellent” [25,26,28,34], while one [18] was rated as “acceptable” based on the items examined. Thus, one article received an “Acceptable” methodological rating, seven were rated “Very Good”, and five were rated “Excellent” (see Table 5).

## 4. Discussion

The menstrual cycle is considered one of the most significant biological rhythms in women, as it causes changes in the body due to hormonal fluctuations [4]. Therefore, the aim of this systematic review was to analyze studies published in the last decade that investigate how these hormonal fluctuations, associated with different phases of the menstrual cycle, affect women’s sports performance, as measured by assessment tests and game-related variables.

Hormonal fluctuations throughout the cycle necessitate an evaluation of sports performance across different phases, which can complicate the interpretation of results. In this review, all studies examined performance parameters or match-related metrics during the follicular phase, particularly the early follicular phase, which coincides with menstruation [20,21,22,25,27,29,30]. The luteal phase was also analyzed in the included studies, with a particular focus on differentiating the mid-luteal phase [22,24,29,30,32] from the premenstrual phase [26,31]. Beato and colleagues [33] highlight that both phases are crucial for analyzing symptom management and hormonal changes.

Match performance metrics indicated better results during the luteal phase, with significant increases in high-intensity bouts, sprints, and high-intensity runs in zone 3 [24]. Supporting these findings, Igoinin and colleagues [30] reported a statistically significant decrease in total distance covered, as well as in moderate- and high-speed runs during the follicular phase. Similarly, a recent meta-analysis [34] explained that performance might decline during the early follicular phase compared to other stages of the cycle, a finding supported by another study [18]. In a study conducted by Arenas-Pareja and colleagues [35] involving professional basketball players, a decline in performance was observed during menstruation and the luteal phase, while the late follicular phase and ovulation showed the highest performance peaks. Specifically, Gasperi and colleagues [28] found improved shooting and rebounding performance in basketball players during the follicular phase compared to the luteal phase, without distinguishing between early and late follicular stages, and reported significant differences in the results.

Regarding performance parameters assessed through evaluation tests, particularly strength indicators, significant differences were found in short-duration, high-intensity runs in a study by Graja [26], which examined explosive lower-limb strength in athletes. A decrease in peak power and reduced neuromuscular efficiency of the vastus lateralis and rectus femoris muscles in maximal voluntary contraction (MVC) after repeated sprint ability (RSA) tests was observed during the premenstrual phase. Additionally, this study [26] reported higher creatine kinase (CK) levels in the blood following repeated sprint efforts (RSEs) and MVC tests during the premenstrual phase, indicating increased muscle damage in athletes during this stage. This could be explained by the hypothesis that higher estradiol (E2) levels during the follicular and luteal phases, compared to the premenstrual phase, act as antioxidants via receptor mechanisms, reducing serum CK levels induced by exercise [19]. Specifically, this study found lower CK levels and improved muscle recovery 96 h post-exercise during the follicular phase compared to the luteal phase [19]. A study by Rodrigues, De Azevedo, and Wharton [36] reported greater maximal strength capacity (1RM) during the late follicular phase compared to menstruation and the late luteal phase, where strength generation capacity declined, consistent with findings from another study [32]. Additionally, some publications [37,38] align with these results, reporting increased muscle strength during the follicular and ovulatory phases compared to the luteal phase. This could be attributed to the role of E2 in the secretion and metabolism of growth hormone (GH), which, due to its anabolic properties, enhances muscle strength [39]. However, an innovative study [40] on CrossFit athletes, evaluated using the Karen protocol (for specific strength), 1RM (maximal strength), and CMJ (explosive strength), found no fluctuations in results related to the menstrual cycle. These findings align with other studies in this review that used jump tests [20,21,25,27,29] and strength assessments such as the half squat [22].

Regarding maximal aerobic capacity, some studies [23,31] reported a decline in aerobic capacity during the follicular phase, with better results during the luteal phase in both the PWC170 test and the Balke maximal test. Conversely, Ross’s study [20] observed higher aerobic capacity during the luteal phase, with greater distances covered in the Yo-Yo Intermittent Endurance Test (Yo-Yo IET). Additionally, the same study showed that three- and five-minutes post-test, blood lactate concentration was higher during the follicular phase than the luteal phase, potentially due to the significantly greater total distance covered in the follicular phase. Furthermore, a recent study [41] contradicted Ross’s [20] findings regarding lactate concentration after a submaximal test, reporting an increase in lactate levels during the luteal phase, consistent with other studies [42]. Berend and colleagues [43] also emphasized that lactate concentration is influenced by an athlete’s nutrition, which should be considered a key study variable when interpreting results.

Regarding flexibility, as assessed by the sit and reach test [25], a significant decrease in flexibility was observed during the early follicular phase, whereas greater flexibility of the hamstrings and lumbar muscles was noted during ovulation. Supporting these findings, other studies have also reported fluctuations in flexibility across different phases of the menstrual cycle [44,45,46]. These variations could contribute to injury risk, such as anterior cruciate ligament (ACL) injuries. It is believed that estrogen affects collagen degradation and synthesis throughout the cycle, influencing biomechanical and anatomical factors specific to female athletes. This hormonal influence may lead to increased knee joint laxity and reduced muscle stiffness, thereby elevating the risk of injury in female athletes [47]. Recent studies are investigating the role of relaxin in ACL integrity, as its concentration progressively increases during ovulation [48]. Additionally, one of the studies included in this review reported a slight improvement in agility, as measured by the V-cut test, during the follicular phase [27].

Furthermore, the studies included in this review not only examined physiological performance but also considered cognitive factors influencing performance and injury rates, as well as subjective variables perceived by athletes. One of the reviewed studies [28] reported a positive effect in both the follicular and luteal phases, showing improved ratings of perceived exertion (RPE) and total quality recovery (TQRpre) in shooting effectiveness during basketball games, ultimately benefiting team performance. These findings are consistent with other studies [49]. Additionally, the results demonstrated that subjective well-being, measured using the Hooper Index [27], was influenced by menstrual phases, with lower well-being values during the early follicular phase and late luteal phase, in accordance with the results observed in numerous other studies [50,51,52,53,54].

## 5. Conclusions

Current research demonstrates that some performance parameters change throughout the menstrual cycle. Flexibility is enhanced during the ovulatory phase, while the luteal phase appears to positively affect both aerobic and anaerobic capacity. In contrast, specific variables like shooting accuracy and the number of rebounds in basketball, are higher during the follicular phase. Additionally, during the menstrual and premenstrual phases, athletes generally report an increase in symptoms with a related reduction grow in perceived performance, negatively impacting certain performance parameters. However, the relationship between other variables, such as maximal and explosive strength, remains unclear and requires further investigation.

The coaching staff should monitor the menstrual cycle of athletes due to the hormonal fluctuations that affect their bodies. This could influence performance parameters and help improve knowledge about the athletes, thereby optimizing their future performance.

## Figures and Tables

**Table 1:** Inclusion and exclusion criteria.

Inclusion Criteria	Exclusion Criteria
- Articles evaluating the potential influence of the menstrual cycle on the performance of female athletes - Studies conducted on eumenorrheic women - Articles analyzing physiological and anthropometric variables throughout the menstrual cycle phases - Articles published between 2013 and 2023 - Articles available in English or Spanish	- Non-original articles - Articles from websites - Articles with only qualitative results (mood, perceived exertion by Borg scale, interviews) - Studies that do not address the research question - Studies with women suffering from menstrual cycle disorders - Case studies, pilot, and preliminary studies - Studies analyzing the effects of contraceptive pills - Studies conducted on active women who are not athletes - Duplicate articles

**Table 2:** Data extraction from included articles.

							Variables Analyzed				
Author, Year and Design of the Study	Study Design	Observation Time	Sport	Category	Sample No.	Average Age	CM Measurement	Phases Analyzed	Parameters and Performance Tests	Subjective Variables	Main Conclusions
Shaktina et al., (2016) [18]	Observational	2 months	Athletics (800 and 1500 m)		13	17-24	Basal body temperature	FF (FM), FOVU and FL (FPM)	PWC Test		FM, FOVU and FPM reduce endurance performance
									4 × 400 m		
Stefanovský et al., (2016) [19]	Randomized	3 months	Judo		8	18.44	Notification from the first day of bleeding and calculation of the phases	FFF and FLM	Wingate		No significant differences were found in the tests performed
									SJFT		
Ross et al., (2017) [20]	Observational	3 months	Soccer	2nd German Division	9	18.6	App registrations and blood samples	FFT and FLM	CMJ		Negative impact of FLM on aerobic capacity
									3 × 30	RPE	
Tounsi et al., (2018) [21]	Observational	1 month	Soccer	High level	11	21.18	Blood tests	FFT, FFF and FL	5JT		Strength and endurance tests showed no interference from CM in sportswomen
									RSA		
Romero-Moraleda et al., (2019) [22]	Randomized	1 month	Triathlon		13	31.1	App registration and urine tests	FFT, FFF and FLM	1RM half-squat		No differences in strength were found during the CM phases
Nabo et al., (2021) [23]	Observational	1 month	Indoor soccer	1st National League	14	24.1	Registration by app	FF and FL	Balke maximal test		Greater maximum aerobic capacity during the luteal phase
Ross et al., (2021) [24]	Observational	4 months	Soccer	1st and 2nd German 15 Divisions	15	23	Records, blood tests and urine tests	FF and FL	Distance and intensity by zones measured by GPS in matches		Greater distances traveled at high intensity in FF
Campa et al., (2021) [25]	Observational	50 days	Soccer	Italian 1st Division	20	23.8	Calendar records	FFT and FOVU	CMJ		A notable influence of CM on flexibility, with greater capacity during ovulation
									20 m		
									Sit and reach		
Graja et al., (2022) [26]	Randomized	30 days	Handball	National League Tunisian	10	22.5	Blood tests, urine tests and records	FF, FL and FPM	MVC	Hooper Index	FPM reduces sprinting ability and strength, with increased muscle damage after the tests
									20 × 5 by bike		
Igoinin et al., (2022) [23]	Observational, longitudinal	3 years	Soccer	French 2nd Division	8	25.7	Registration by app	FFT, FFF and FLM	Match statistics (Total distance and speeds reached)		Longer total distance traveled, and moderate and high speed in FL
Sánchez et al., (2022) [27]	Observational	3 months	Soccer	Regional	12	16.18	Records by app	FFT, FFF and FL	40 m	Hooper Index	Reduction in subjective well-being during FM and FL
									V-cut,		
									Hop test (unipodal and bipodal) and SJ		
Gasperi et al., (2023) [28]	Descriptive, retrospective	14 weeks	Basketball	1st Lithuanian Division	11	20.5	Notification from the first day of bleeding and calculation of the phases	FF and FL	Match stats (PIR, REB, eFG%, TO, PL min) * and factors, game contexts	RPE and TQRpre	Better rebounds and shooting effectiveness during FF, with correlation of Subjective variables with shooting in matches
Morenas-Aguilar et al., (2023) [29]	Observational	4 months	Handball	Regional	8	19.8	Urine analysis and calendar	FFT, FFF and FLM	CMJ and pitching velocity	SAM scale	CM does not affect the performance variables of athletes
										VAS scale	

Abbreviations: CM (menstrual cycle); FF (follicular phase); FM (menstrual phase); FOVU (ovulatory phase); FL (luteal phase); FPM (pre-menstrual phase); FFF (final follicular phase); FLM (mid-luteal phase); FFT (early follicular phase); Test PWC (physical working capacity), SJFT (special judo fitness test), CMJ (countermovement jump), RPE (rate of perceived exertion); TQRpre (total quality recovery); 5JT (5-jump test), RSA (repeated sprint ability), MVC (maximum voluntary contraction), SJ (squat jump); PIR (performance index rating), REB (rebounds), eFG% (effective field goal percentage), TO (turnovers), PL min (player load per minute), SAM (self-assessment manikin), VAS (visual analogue scale).

**Table 3:** Analysis of the results of studies in collective sports.

Authors	Sports	Principal Results of Performance Test	Principal Results of Psychological Variables
Ross et al., (2017) [20]	Soccer	↔ CMJ ↔ 3 × 30 sprint ↓ Total distance covered in Yo-Yo IET on LF * ↑ FC pre Yo-Yo test in LF * ↑ 3-5′ lactate post Yo-Yo test in FF *	↔ RPE in MC
Tounsi et al., (2018) [21]	Soccer	↔ 5JT y RSA between phases ↑ In the tests (5JT and RSA) when carried out in the afternoon * ↑ YYIRT1	
Nabo et al., (2021) [23]	Futsal	↑ VO2Max in FL compared to FF in Balke Maximal Test * ↑ Duration in Balke Test in LF *	
Ross et al., (2021) [24]	Soccer	↑ Maintenance in zone 3 and high-intensity races in LF compared to FF * ↑	
Campa et al., (2021) [25]	Soccer	↔ CMJ ↔ 20 m ↑ Flexibility in the FO compared to the FF in the Sit and Reach test *	
Graja et al., (2022) [26]	Handball	↔ % dec sprint in FF and FL in RSA * ↓ %dec in sprint in FF tan in FPM in RSA * ↓ PP in FPM tan in FF and FL in RSA * ↓ PP in two final *sprints* in RSA in FL than in FF * ↑ MVC in FF post RSA than in FPM and FF * ↓ NME in vastus lateralis and rectus femoris in FPM post RSE than in FF and FL * ↓ MDF in vastus lateralis and rectus femoris in FPM post RSE than in FF and FL *	↔ During the three phases of CM in the Hooper Index (stress, sleep, pain, and fatigue)
Igonin et al., (2022) [30]	Handball	↓ Total distance in FF than in FL * ↓ Moderate speed in FF tan in FL * ↓ High speed in FF than in FL * ↑ Number *sprints* in FL *	
Sánchez et al., (2022) [27]	Soccer	↔ Hop test, SJ ↔ 40 m ↔ V-cut	↓ Subjective well-being in FM and FL compared to FF in the Hooper Index * ↑ Fatigue in FM and LF compared to FF
Gasperi et al., (2023) [28]	Basketball	↑ EFG% in FF than in LF * ↑ REB in FF than in LF *	↑ RPE and TQRpre are related to higher EFG% with better shooting in FF and LF *
Morenas-Aguilar et al., (2023) [29]	Handball	↔ CMJ ↔ Launch speed	↔ Mood and perceived pain throughout the phases evaluated by the SAM and VAS scales

Abbreviations: MC (menstrual cycle); CMJ (countermovement jump); LP (luteal phase); 5JT (5-jump test); RSA (repeated sprint ability); VO2Max (maximum oxygen consumption); FP (follicular phase); DEC (deceleration); PM (premenstrual phase); PP (peak power); MVC (maximum voluntary contraction); NME (neuromuscular efficiency); MDF (median frequency); SJ (squat jump); eFG% (effective field goal percentage); REB (rebounds); RPE (rate of perceived exertion); TQRpre (total quality recovery); SAM (self-assessment manikin); VAS (visual analogue scale). Symbols: “↑” significant increase in capacity compared to other phases of the menstrual cycle, “↓” significant decrease in capacity compared to other phases of the menstrual cycle, “↔” no significant differences in capacity between phases, “*” statistically significant results (*p* < 0.05).

**Table 4:** Analysis of results from studies on individual sports.

Authors	Sports	Principal Results Of Performance Test	Principal Results of Psychological Variables
Shakhlina et al., (2016) [31]	Athletics	↓ Performance in series of 4 × 400 m in FM and FPM * ↑ PWC170 in FL compared to FF and FOVU * ↑ Blood lactate post 4 × 400 m in FM, FPM, and FOVU * ↑ FM, FPM, and FOVU in the average HR post 4 × 400 *	Not include the analysis of subjective variables
Štefanovský et al., (2016) [32]	Judo	↔ Test wingate ↔ SJFT ↑ In the first 15 seconds of SJFT in FLM *	
Romero-Moraleda et al., (2019) [22]	Triathlon	↔ Estimates of 20, 40, 60, and 80% of 1RM in half squat in FF and FL ↔ Speed estimates 1RM half squat in FF and FL ↔ Power estimates 1RM half squat in FF and FL	

Abbreviations: MP (menstrual phase); PM (premenstrual phase); PWC (physical working capacity); LP (luteal phase); FP (follicular phase); OP (ovulatory phase); HR (heart rate); SJFT (special judo fitness test). Symbols: “↑” significant increase in capacity compared to other phases of the menstrual cycle, “↓” significant decrease in capacity compared to other phases of the menstrual cycle, “↔” no significant differences in capacity across phases, “*” statistically significant results (*p* < 0.05).

**Table 5:** Critical review results of McMaster.

References		Shakhlina et al., (2016) [31]	Štefanovský et al., (2016) [32]	Ross et al., (2017) [20]	Tounsi et al., (2018) [21]	Romero-Moraleda et al., (2019) [22]	Nabo et al., (2021) [23]	Ross et al., (2021) [24]	Campa et al., (2021) [25]	Graja et al., (2022) [26]	Igonin et al., (2022) [30]	Sánchez et al., (2022) [27]	Gasperi et al., (2023) [28]	Morenas-Aguilar et al., (2023) [29]	
	1	1	1	1	1	1	1	1	1	1	1	1	1	1	13
	2	1	1	1	1	1	1	1	1	1	1	1	1	1	13
	3	0	0	1	0	0	0	1	0	0	0	1	0	0	3
	4	0	1	1	1	1	0	1	1	1	0	0	1	0	8
	5	1	1	1	1	1	1	1	1	1	1	1	1	1	13
	6	0	0	1	0	0	0	0	1	0	0	1	1	1	5
**I**	7	1	1	1	1	1	1	1	1	1	1	1	1	1	13
**T**	8	1	1	1	1	1	1	1	1	1	1	1	1	1	13
**M**	9	0	1	1	1	1	1	1	1	1	1	1	1	1	12
**S**	10	1	1	1	1	1	1	1	1	1	1	1	1	1	13
	11	1	1	1	1	1	1	1	1	1	1	1	1	1	13
	12	1	1	1	1	1	1	1	1	1	1	1	1	1	13
	13	1	1	1	1	1	1	1	1	1	1	1	1	1	13
	14	0	1	1	1	1	1	1	1	1	1	1	1	1	12
	15	1	1	1	1	1	1	1	1	1	1	1	1	1	13
	16	1	1	1	1	1	1	1	1	1	1	1	1	1	13
**T**		11	14	16	14	14	13	15	15	14	13	15	15	14	

In the table of results of the McMaster critical review, the questions on the form are abbreviated with numbers from 1 to 16. The answers to the questions are evaluated with a 1 if the answer is “Yes” and a 0 if the answer is “No”.

## Data Availability

The data presented in this study are available on request from the corresponding author.
